# Mitochondrial translocator protein deficiency exacerbates pathology in acute experimental ulcerative colitis

**DOI:** 10.3389/fphys.2022.896951

**Published:** 2022-08-19

**Authors:** Isabel A. Jimenez, Allison P. Stilin, Kanako Morohaku, Mahmoud H. Hussein, Prasanthi P. Koganti, Vimal Selvaraj

**Affiliations:** ^1^ Department of Animal Science, College of Agriculture and Life Sciences, Cornell University, Ithaca, NY, United States; ^2^ Department of Molecular and Comparative Pathobiology, The Johns Hopkins University School of Medicine, Baltimore, MD, United States; ^3^ School of Science and Technology, Institute of Agriculture, Shinshu University, Nagano, Japan

**Keywords:** colon, inflammation, mast cell, mitochondria, intestinal epithelium

## Abstract

In human patients and animal models of ulcerative colitis (UC), upregulation of the mitochondrial translocator protein (TSPO) in the colon is consistent with inflammation. Although the molecular function for TSPO remains unclear, it has been investigated as a therapeutic target for ameliorating UC pathology. In this study, we examined the susceptibility of *Tspo* gene-deleted (*Tspo* ^*−/−*^) mice to insults as provided by the dextran sodium sulfate (DSS)-induced acute UC model. Our results show that UC clinical signs and pathology were severely exacerbated in *Tspo* ^*−/−*^ mice compared to control *Tspo* ^*fl/fl*^ cohorts. Histopathology showed extensive inflammation and epithelial loss in *Tspo* ^*−/−*^ mice that caused an aggravated disease. Colonic gene expression in UC uncovered an etiology linked to precipitous loss of epithelial integrity and disproportionate mast cell activation assessed by tryptase levels in *Tspo* ^*−/−*^ colons. Evaluation of baseline homeostatic shifts in *Tspo* ^*−/−*^ colons revealed gene expression changes noted in elevated epithelial *Cdx2*, mast cell *Cd36* and *Mcp6*, with general indicators of lower proliferation capacity and elevated mitochondrial fatty acid oxidation. These findings demonstrate that intact physiological TSPO function serves to limit inflammation in acute UC, and provide a systemic basis for investigating TSPO-targeting mechanistic therapeutics.

## 1 Introduction

Overexpression of the mitochondrial translocator protein (TSPO) in the colon is a pathological feature observed in human ulcerative colitis (UC) (Ostuni et al., 2010), a form of inflammatory bowel disease (IBD). Consistent with human pathology, rodent models of UC have confirmed that TSPO upregulation in the colon is characteristic of UC (Ostuni et al., 2010). This finding is supported by studies investigating TSPO-binding drugs as radioligands for positron emission tomography (PET)-based diagnostic imaging; it has been concluded that TSPO detection using these probes provides a non-invasive method to evaluate the level and localization of colon inflammation (Bernards et al., 2015; Kurtys et al., 2017). High expression of TSPO in chronic UC has also been linked to an increased risk of colorectal cancers (Katz et al., 1988, 1990). Association between TSPO and inflammation is not only restricted to observations made in the colon; TSPO upregulation has been reported in conditions that induce neuroinflammation (Airas et al., 2015), cardiovascular inflammation (Fujimura et al., 2008), and rheumatoid arthritis (Narayan et al., 2018). Indicative of an active function and/or etiology for TSPO involvement in these pathologies, preclinical models have demonstrated that a variety of TSPO-binding drugs are efficacious as therapeutics in ameliorating inflammation. Protective effects of TSPO-targeting therapies have been reported for disorders such as multiple sclerosis (Daugherty et al., 2013), brain injury after intracerebral hemorrhage (Li et al., 2017), diabetic neuropathy (Giatti et al., 2009), autoimmune arthritis (Waterfield et al., 1999), and Alzheimer’s disease (Barron et al., 2013). As the etiology and pathophysiology of UC remains to be fully defined, TSPO has been considered as a therapeutic target and investigated for reducing both stress and colon inflammation.

Of direct relevance to therapeutic efficacy in UC, the TSPO-binding drug ONO-2952 (1-[(1S)-1-(4-chloro-2-methoxyphenyl)-5-fluoro-1,9-dihydrospiro [β-carboline-4,1’- cycloprop ane]-2(3H)-yl]ethenone) has been under development as a treatment for diarrhea-predominant irritable bowel syndrome (IBS-D) (Ono Pharma United States Inc.; NCT01844180, ClinicalTrials.gov). IBS-D patients have significantly higher levels of pro-inflammatory cytokines indicative of gut inflammation (Rana et al., 2012). ONO-2952 showed high selectivity in binding to TSPO, and could promote anti-stress effects by inducing a variety of protective responses (Mitsui, 2012; Mitsui et al., 2015). In preclinical rat models, ONO-2952 attenuated stress-induced defecation and rectal hyperalgesia (Kawahara et al., 2018). In the Phase 2 trial, ONO-2952 showed evidence for clinical efficacy with promising trends supporting its consideration as a potential treatment (Whitehead et al., 2017). Similarly, flunitrazepam, albeit a non-selective TSPO-binding drug, could protect the intestinal mucosa against DSS-induced damage (Ostuni et al., 2010). However, actions of other TSPO-binding drugs have not been consistent; PK11195 and Ro5-4864 were observed to enhance initial severity of colonic erosion, but accelerate recovery (Ostuni et al., 2010). Because the molecular function of TSPO remains to be elucidated (Selvaraj and Stocco, 2015; Selvaraj and Tu, 2016), TSPO-binding drugs cannot be confirmed as agonists or antagonists based on current understanding of target effects, potentially accounting for these differences in TSPO-targeting therapeutic efficacy.

First identified as a “receptor” showing high affinity binding to diazepam and its derivative 4’chlorodiazepam (Braestrup and Squires, 1977), the protein sequence of TSPO is highly conserved across microbes, plants and animals (Selvaraj and Stocco, 2015). Although pharmacology linked to TSPO-binding has indicated a variety of potential functions (Gavish et al., 1999), development and utilization of TSPO gene-deleted (*Tspo* ^*−/−*^) models have ruled out its involvement in key purported pharmacological functions such as induction of *de novo* steroidogenesis (Tu et al., 2014a; Banati et al., 2014; Morohaku et al., 2014b; Wang et al., 2016), mitochondrial permeability transition (Šileikyte et al., 2014), and heme biosynthesis (Zhao et al., 2016). Phenotypic observations in *Tspo* ^*−/−*^ models point to TSPO’s functional involvement in lipid/energy metabolism (Leduc et al., 2011; Thompson et al., 2013; Tu et al., 2016; Liu et al., 2017; Kim et al., 2019; Koganti and Selvaraj, 2020). While such metabolic modulation might be relevant to certain TSPO over-expressing colon cancers (Katz et al., 1990; Maaser et al., 2005; Königsrainer et al., 2007), the precise mechanism of TSPO function in this context remains to be defined (Selvaraj and Stocco, 2015; Betlazar et al., 2020).

Given the considerable relevance of TSPO in the context of etiology, pathogenesis and therapeutic interventions for UC, we investigated *Tspo* ^*−/−*^ mice as a loss-of-function preclinical model to study the relevance of TSPO in homeostasis and TSPO-mediated mechanisms in UC. Such genetic studies provide an opportunity to delineate gene function and distinguish this from the non-specific actions prominently seen with different TSPO-binding drugs (Bordet et al., 2007; Li et al., 2008; Luc Do Rego et al., 2015; Tu et al., 2015; Singh et al., 2020). Our results identify a clear protective function for TSPO expression in colitis, deficiency of which results in exacerbated colonic inflammatory pathology.

## 2 Materials and methods

### 2.1 *Tspo* gene-deleted mice

Generation and validation of *Tspo* ^*−/−*^ mice has been previously described (Morohaku et al., 2014a; Tu et al., 2014b; Zhao et al., 2016). Breeding colonies for *Tspo* ^*fl/fl*^ and *Tspo* ^*−/−*^ mice were propagated in the C57BL/6 background. Both male and female mice (*Mus musculus*) (8–10 weeks of age) were used in this study. Animals were maintained in accordance with the National Research Council Guide for the Care and Use of Laboratory Animals. All experiments reported in this manuscript were approved by The Institutional Animal Care and Use Committee of Cornell University.

### 2.2 Translocator protein detection by Western blots

Detection of TSPO in Western blots was performed as previously described (Morohaku et al., 2013). In brief, colon samples were homogenized using a bead beater (Biospec Products), boiled in Laemmli sample buffer (Laemmli, 1970), and total protein was quantified using a bicinchoninic acid (BCA) colorimetric assay. Twenty micrograms of protein was then separated by SDS-PAGE, transferred to PVDF membranes and immunoblotted for the presence of TSPO. In brief, membranes were blocked using 5% non-fat dry milk in tris buffered saline containing 0.2% Tween 20 (TBST) and incubated with rabbit anti-TSPO monoclonal antibody (1:1,000; Abcam, EPR5384) and control mouse anti-actin affinity purified polyclonal antibody (1:1,000; LI-COR). Detection was performed by incubation with IRDye® 800 conjugated goat anti-rabbit IgG and IRDye® 700 conjugated goat anti-mouse IgG secondary antibodies followed by imaging using a laser fluorescence scanner (LI-COR) for simultaneous detection of the two emission wavelengths in the same blot.

### 2.3 Experimental acute ulcerative colitis

Induction of acute UC was performed using a dextran sodium sulfate (DSS) model (Okayasu et al., 1990). This model involved the oral administration of DSS (36–50 kDa, MP Biomedicals) that compromises mucosal barrier function in treated mice. For the experimental treatment groups, *Tspo* ^*fl/fl*^ and *Tspo* ^*−/−*^ mice received DSS dissolved in autoclaved drinking water to a 2.5% solution (w/v) in negative pressure bottles *ad libitum* for 7 days. For the control groups, *Tspo* ^*fl/fl*^ and *Tspo* ^*−/−*^ mice continued to receive regular drinking water for the same time period. For both the treatment and control groups of both genotypes, phenotypic recordings were performed daily. This included body weight measurements, stool consistency recordings, and scoring the phenotypic severity. Criteria used for stool scoring system were: 0—Normal appearance, 1—Observation of soft feces or pink color in anus, 2—Mild rectal bleeding, or blood observed only in feces, 3—Moderate rectal bleeding, 4—Severe rectal bleeding. Criteria used for posture scoring system were: 0—Normal appearance, 1—Slight hunched posturing, 2—Moderate hunching, reduced mobility, 3—Severe hunching, very little mobility. Criteria used for scoring phenotypic severity were: 1—observation of soft feces or pink color in anus, indicative of irritation, 2—blood observed in feces, or mild rectal bleeding, 3—moderate rectal bleeding, 4—severe rectal bleeding. The phenotypic clinical scoring data was utilized to generate an average stool score and average posturing score for each group. For sample collection, mice were euthanized for tissue collection for histopathology at the end of the 7-day treatment period; separate experimental runs were performed for collections at 0 (baseline, prior to any treatment), 4- and 7-day treatment periods for gene expression analyses.

### 2.4 Gross pathology and tissue collection

At the end of each study, colons (the complete colorectal region) were collected after euthanasia and flushed with ice-cold phosphate-buffered saline from proximal to distal to remove fecal matter. Their length was measured using digital calipers. Colons were then dissected along the long axis to open up the lumen for gross pathological examination of the mucosa (hyperemia, petechial hemorrhage, punctate to broad-based ulcers of various sizes). Tissues were then either fixed for histopathology, or snap frozen in liquid nitrogen and held at -80°C before further processing for gene expression assays.

### 2.5 Histopathology

Full lengths of the colons were prepared for fixation by rolling from distal to proximal as a “Swiss roll” with the mucosa facing inside, a previously described method to examine the full length of tissue in sections (Whittem et al., 2010). The rolled tissue was fixed in place with 4% formaldehyde in phosphate buffered saline for 48 h. Tissues were then transferred to 70% ethanol for 24 h and taken through steps of dehydration and embedded in paraffin. Sections (4 µm thick) were cut using a microtome, and hematoxylin and eosin staining was performed to visualize tissue morphology using standard histological methods, also previously described (Morohaku et al., 2013; 2014a). Slides were studied under a light microscope (DM1000-LED, Leica), and images were acquired using a HD camera (ICC50HD, Leica); full slides were also scanned at ×40 magnification using the Aperio CS2 Scanscope (Leica). Image analysis was performed to quantify proximal distance of ulcerated tissue showing epithelial loss. In brief, lengthwise measurements of colonic epithelial pathology (from distal to proximal) and total colon length were made from scanned histopathology images after calibration using Adobe Illustrator. These values were used to generate a ratio of affected to total colon length for comparison between the groups.

### 2.6 Translocator protein immunolocalization

After deparaffinization and rehydration (as above), sections were subjected to antigen retrieval using 0.01 M citrate buffer. Non-specific binding was blocked using 5% normal goat serum, then samples were incubated with anti-TSPO antibody (1∶200; Abcam, EPR5384) in 1% BSA in PBS overnight at 4°C. After incubation, slides were washed in PBS and incubated with pHRP-conjugated anti-rabbit secondary antibody, and processed using the DAB chemistry to visualize positive staining as previously described (Morohaku et al., 2013). To highlight morphology, slides were counterstained for a weak hematoxylin background. Images were acquired as described for histopathology.

### 2.7 Gene expression assays

Total RNA was extracted from the colon (distal 1.5 cm) from *Tspo* ^−/−^ and *Tspo* ^*fl/fl*^ treatments and controls using TRIzol^TM^ Reagent (Life Technologies), after tissue disruption (Bead beater, Biospec). Extracted total RNA was further purified using lithium chloride (Viennois et al., 2013), as we confirmed that DSS inhibited reverse transcription for qPCR amplification of cDNA from tissues as previously described (Kerr et al., 2012). In brief, 0.1 volume of 8M LiCl was added to total RNA, and incubated at −20°C for 2 h. After incubation, samples were immediately centrifuged for 30 min to pellet total RNA at 14,000 x g at 4°C. After discarding the supernatant, the pellet was washed with 70% ethanol and air dried before resuspension in RNAse-free water. RNA concentration was quantified using a spectrophotometer (NanoPhotometer® Pearl, Implen). Reverse transcription of 1 µg of total RNA was carried out using Multiscribe^TM^ reverse transcriptase (Thermo Fischer Scientific). Validated TaqMan™ gene expression assays (Applied Biosystems) and intron-spanning primers for SYBR® green detection were used for quantitative PCR to estimate levels of *Krt18, Cd34, Mcp6, Cd11c, F4/80, Mcp1, Tnfα, Lgr5, Ascl2, Cdh1, Ndrg2, cMyc, Ccnd1, Cdx2, Socs3, Cpt1a, Acadm, Acadl, Hadha, Cd36, FpnI, Hmox,* and *Nrf2.* All expressions were normalized to an internal control gene *Gapdh* or *Tbp*. Primer probe sets and sequences are provided in Supplementary Table S1. Relative fold-change between *Tspo* ^−/−^ and *Tspo* ^*fl/fl*^ mice was calculated using the 2^−ΔΔCt^ method (Livak and Schmittgen, 2001).

### 2.8 Statistics

Means for the posturing and stool scores were compared between genotypes using a Student’s t-test. Colon length and daily body weight changes were compared using repeated measures ANOVA and individual timepoints were compared using Student’s two-tailed t-tests. Histopathological colon length was analyzed by first calculating a ratio of affected to normal colonic tissue for each mouse after 7 days of DSS treatment, and comparing the ratios from *Tspo* ^*−/−*^ and *Tspo* ^*fl/fl*^ groups using a two-proportions z-test. Gene expression differences between *Tspo* ^*−/−*^ and *Tspo* ^*fl/fl*^ groups at the different timepoints were compared using Student’s two-tailed t-tests. For all analyses *p* < 0.05 was considered significant.

## 3 Results

### 3.1 Exacerbated ulcerative colitis clinical signs and gross pathology in *Tspo* ^−/−^ mice

**FIGURE 1:**
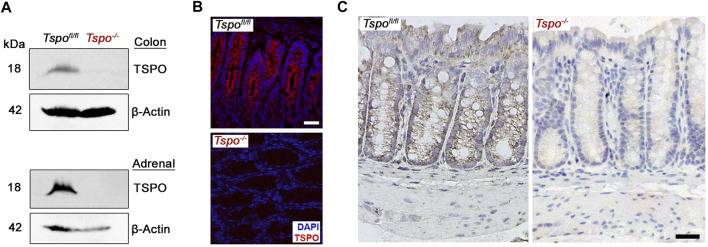
TSPO expression in *Tspo* ^*fl/fl*^ and *Tspo* ^−/−^ colons. **(A)** Western blot showing specific recognition of TSPO in *Tspo* ^*fl/fl*^ but not in *Tspo* ^−/−^ colons; adrenals that show high expression of TSPO are presented for comparison. In the same lanes, β-actin detection was used to indicate protein loading (control). **(B–C)** Immunofluorescence and immunohistochemistry images showing TSPO expression in *Tspo* ^*fl/fl*^ but not in *Tspo* ^−/−^ colons. Expression of TSPO can be observed localized to the mucosal layer but not in the submucosal or muscular layers of the colon wall. (Scale bar 30 µm).

**FIGURE 2:**
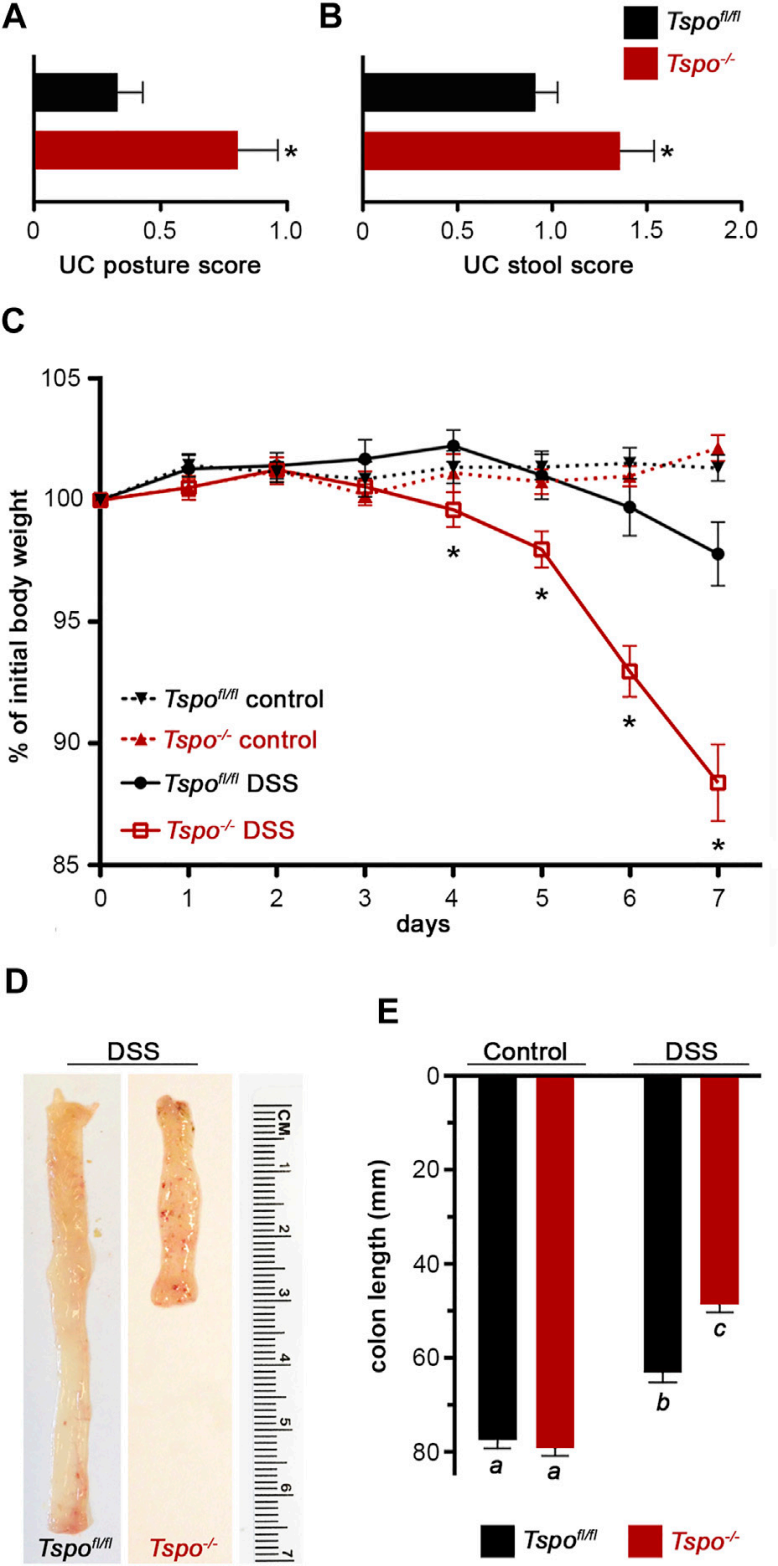
Exacerbated clinical signs and gross pathology of UC in *Tspo* ^−/−^ mice. **(A)** Severity of UC assessed by posturing scoring, days 5–7 of DSS in *Tspo* ^−/−^ and *Tspo* ^*fl/fl*^ mice (*n* = 17 per group; *p* = 0.0053). Scoring: 0, normal behavior; 1, mild hunched posture; 2, hunched posture and reduced movement. **(B)** Severity of UC assessed by stool score, days 3–7 of DSS in *Tspo* ^−/−^ and *Tspo* ^*fl/fl*^ mice (*n* = 17 per group; *p* = 0.03). Scoring: 0, normal stool; 1, soft feces, pink anus; 2, bloody feces, mild rectal bleeding; 3, moderate rectal bleeding; 4, diarrhea, severe rectal bleeding. **(C)** Body weight changes during UC in *Tspo* ^−/−^ mice indicated a significantly higher weight loss compared to *Tspo* ^*fl/fl*^ mice. Temporal changes over the 7-day DSS treatment graphed as percentage of initial body weight in *Tspo* ^−/−^ and *Tspo* ^*fl/fl*^ mice (n = 21 per group; ANOVA *p*=<0.0001). Body weights at Day 4 (*p* = 0.0328), Day 5 (*p* = 0.0193), Day 6 (*p* < 0.0001), and Day 7 (*p* < 0.0001), were significantly different. **(D)** Representative images of colons from *Tspo* ^*fl/fl*^ and *Tspo* ^−/−^ mice after DSS-induced colitis, showing differences in length and the presence of petechiation (red hemorrhagic spots) on the mucosal surface indicative of inflammatory severity. **(E)** Average colon lengths which correlate with severity of UC was significantly shorter in *Tspo* ^−/−^ mice than *Tspo* ^*fl/fl*^ mice after 7 days of DSS treatment (*n* = 16–21 per group; *p* < 0.0001).

Deletion of TSPO was confirmed in *Tspo* ^−/−^ mice used in these studies using both Western blots and immunolocalization in the colon (Figure 1); expression of TSPO was observed in the epithelium, with noted absence in submucosal and muscular layers. In *Tspo* ^−/−^ mice, clinical signs and pathology after DSS-induced UC were significantly more severe compared to *Tspo* ^*fl/fl*^ mice. *Tspo* ^−/−^ mice with UC exhibited significantly worse external clinical signs (phenotypic scores: posture and stool) compared to *Tspo* ^*fl/fl*^ mice (Figures 2A,B). There was a significantly higher degree of rectal bleeding and diarrhea, decreased movement, and hunched posturing observed in *Tspo* ^−/−^ mice. Body weight loss, a well-studied indicator of disease severity in DSS-induced UC, was significantly higher from days 4 through 7 in *Tspo* ^−/−^ mice compared to *Tspo* ^*fl/fl*^ mice (Figure 2C). On postmortem gross pathology, *Tspo* ^*−/−*^ mice exhibited subjectively more petechiae, ecchymoses, and ulceration of the colonic mucosa compared to *Tspo* ^*fl/fl*^ mice (Figure 2D). *Tspo* ^−/−^ mice also exhibited significantly greater colonic shortening, indicative of the severity of colitis, compared to *Tspo* ^*fl/fl*^ mice (Figure 2E). There was no significant difference in colon lengths between male and female mice within their respective cohorts.

### 3.2 Colon histopathology indicated exacerbated ulcerative colitis in *Tspo* ^−/−^ mice

**FIGURE 3:**
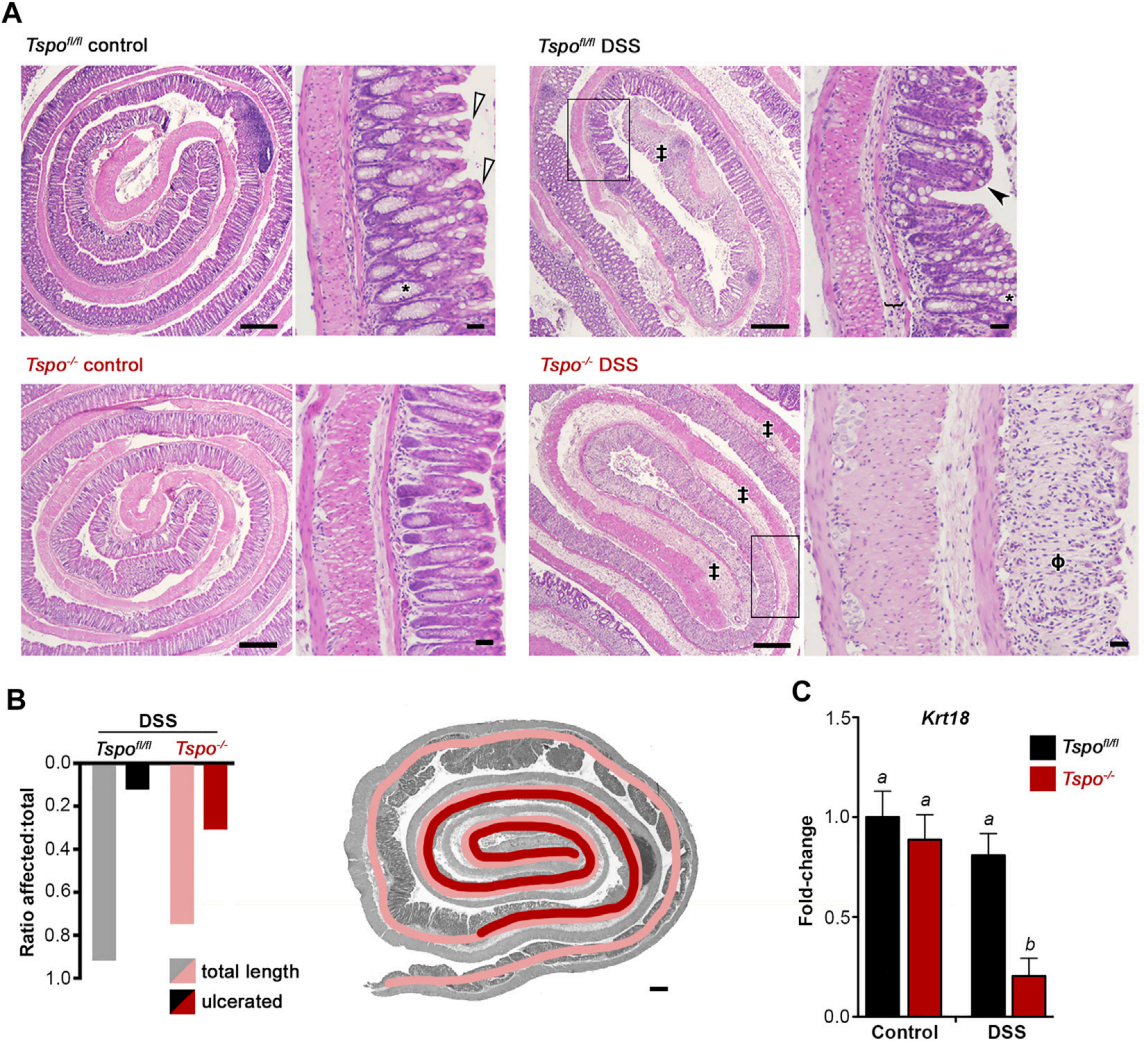
Histopathology and epithelial loss indicate severe inflammation with UC in *Tspo* ^−/−^ mice. **(A)** Histopathological images showing colon rolls (clockwise: inside distal to outside proximal), from control colons and 7-day DSS-induced UC colons. Representative images are shown for both *Tspo* ^−/−^ and *Tspo* ^*fl/fl*^ colons. Control *Tspo* ^−/−^ and *Tspo* ^*fl/fl*^ colons were histologically indistinguishable. Normal morphology consists of basally-located goblet cells (*), a narrow submucosa and thin muscularis mucosa, cuboidal to columnar mature absorptive epithelial cells (white arrowhead). In both UC cohorts, the pathology was most severe at the distal colon, consistent with the pathogenesis of UC. DSS-treated *Tspo* ^*fl/fl*^ animals exhibited segmental moderate UC, with the distal colon demonstrating moderate edema, loss of crypt and goblet cells, moderate to severe inflammation, and erosion and ulceration of the colonic epithelium (‡). In the mid-colon (black box), *Tspo* ^*fl/fl*^ colons showed less severe pathology, characterized by mild submucosal edema (bracket), and mild mononuclear inflammation. The mucosa displays shortened, immature epithelial cells (black arrowhead). Partial loss of the colonic crypts is present, as well as goblet cell hyperplasia and altered distribution of goblet cells towards the apex of the crypts. DSS-treated *Tspo* ^−/−^ animals developed more diffuse UC, affecting the entirety of the distal colon and extending into the mid-colon (black box) and proximal colon. These animals exhibited severe, diffuse colonic edema, extensive crypt and goblet cell loss (ϕ), muscular hypertrophy, epithelial ulceration and erosion, and severe diffuse mononuclear inflammation. (Low magnification scale bar: 300 μm; high magnification scale bar: 30 µm). **(B)** In quantitating the regional degree of colonic epithelial ulceration and erosion due to UC, the ratio of affected:total colon length demonstrated significantly greater damage in *Tspo* ^−/−^ colons than *Tspo* ^*fl/fl*^ colons (0.44 vs. 0.17 respectively; *p* = 0.0039, Z = 2.885, *n* = 9 per group). Representative histopathology image with an overlay of color-coded measurement lines is shown to demonstrate this quantification of disease extent. **(C)** Expression of *Krt18*, an intestinal epithelial marker, in the distal colon of *Tspo* ^−/−^ and *Tspo* ^*fl/fl*^ mice at baseline and at day-7 of DSS-induced UC; significant down-regulation of *Krt18* indicative of epithelial loss is observed only with UC in *Tspo* ^−/−^ colons (*n* = 7 per group; *p* = 0.0011).

Histopathological lesions were consistently more severe after DSS-induced UC in *Tspo* ^−/−^ mice compared to *Tspo* ^*fl/fl*^ mice. Extremely severe colonic pathologies were noted with UC in *Tspo* ^−/−^ mice compared to *Tspo* ^*fl/fl*^ mice (Figure 3A). These included diffuse edema, immune infiltrations, erosion and ulceration of the epithelium, and loss of crypt and goblet cells; progression of ulceration occurred continuously from distal to proximal and correlated with severity of disease. In evaluating the proximal extent of epithelial damage as an indicator of disease severity between the two genotypes, lengthwise measurements of colonic epithelial damage (from distal to proximal) indicated a substantial increase in ulcerative pathology in *Tspo* ^−/−^ mice compared to *Tspo* ^*fl/fl*^ mice (Figure 3B). Based on the ratio of affected:total colon length as an indicator of disease severity, *Tspo* ^−/−^ mice demonstrated a much more proximal extent of epithelial damage (ratio = 0.44), while colonic pathology was limited to the distal colon in *Tspo* ^*fl/fl*^ mice (ratio = 0.17). Based on evaluation of *Krt18* gene expression as a quantitative indicator of colonic epithelial cells, *Tspo* ^−/−^ mice showed significantly more severe epithelial loss (4-fold) following DSS-induced UC treatment compared to *Tspo* ^*fl/fl*^ mice (Figure 3C). This finding indicated a substantial structural loss of epithelial barrier integrity in *Tspo* ^−/−^ mice compared to *Tspo* ^*fl/fl*^ mice, potentially increasing microbial infiltration and exacerbating associated pathologies.

### 3.3 Differences in inflammatory gene expression of *Tspo* ^−/−^ colons

**FIGURE 4:**
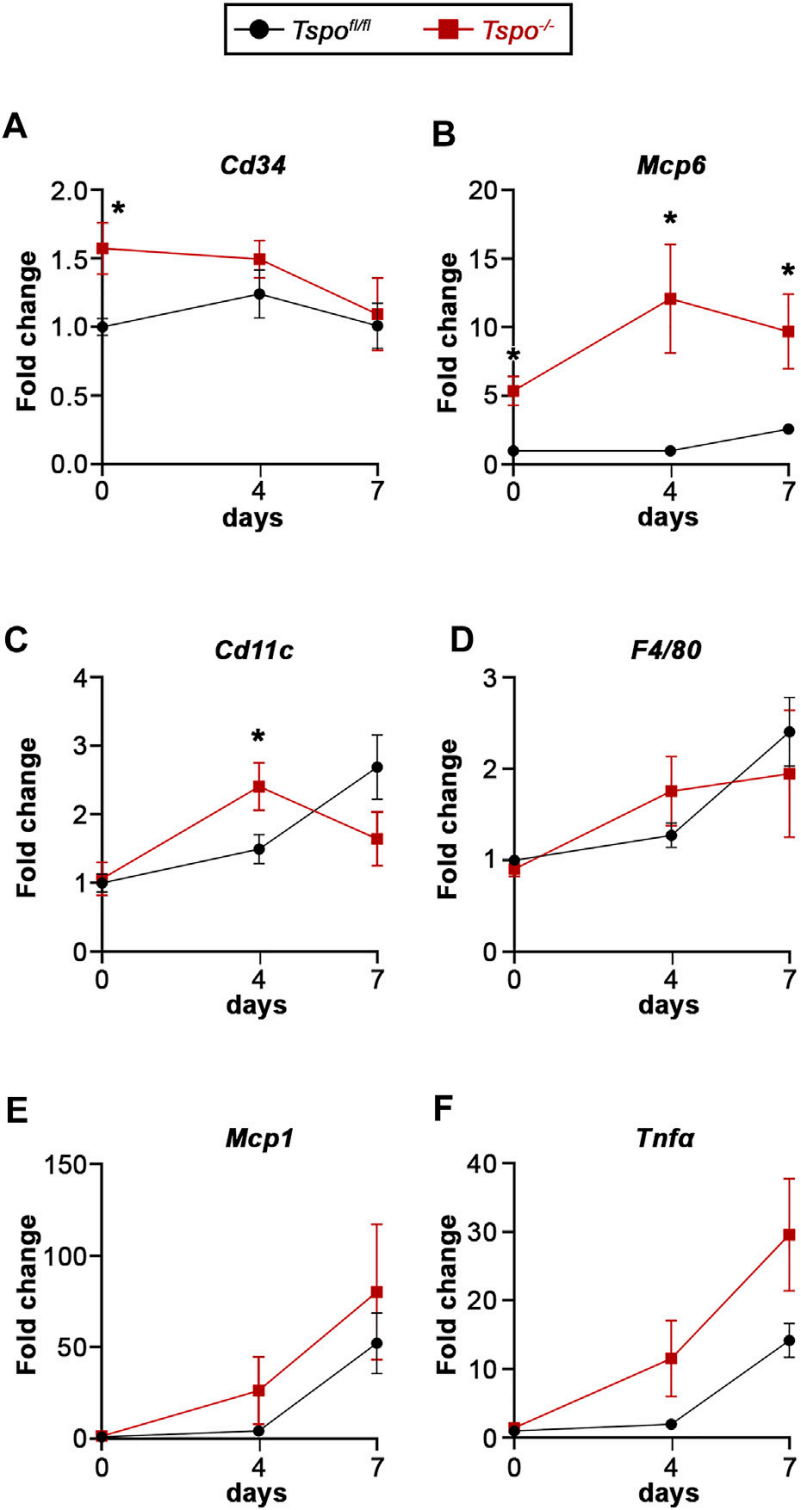
Gene expression indicative of inflammation with UC in *Tspo* ^−/−^ and *Tspo* *^fl/fl^* mice. **(A)** Expression of *Cd34*, a surface marker for mast cells, was significantly higher at baseline (day-0) in *Tspo* ^−/−^ colons compared to *Tspo* *^fl/fl^* colons (*p* = 0.0077), but not at the later UC timepoints. **(B)** Expression of *Mcp6*, a mast cell tryptase, was significantly up-regulated at baseline (*p* = 0.0010) and at all UC timepoints in *Tspo* ^−/−^ colons compared to *Tspo* *^fl/fl^* colons (*p* ≤ 0.0111). **(C)** Expression of *Cd11c*, a dendritic cell transmembrane protein, showed significant up-regulation at day-4 of UC in *Tspo* ^−/−^ colons compared to *Tspo* *^fl/fl^* colons (*p* < 0.0392). **(D)** Expression of *F4/80*, a surface adhesion protein also observed in macrophages, appeared up-regulated in UC but not significantly different between the two genotypes. **(E)** Expression of *Mcp1*, a macrophage activation marker, appeared up-regulated in UC, but not significantly different between the two genotypes. **(F)** Expression of *Tnfα*, a mast cell- and macrophage-released proinflammatory cytokine, was up-regulated with progression of UC, with significant increases between baseline and day-7 in both *Tspo* ^−/−^ (*p* = 0.0089) and *Tspo* *^fl/fl^* (*p* = 0.0002) colons. Although there was a trend showing relative upregulation in *Tspo* ^−/−^ colons compared to *Tspo* *^fl/fl^* colons at day-4 (*p* = 0.0652) and day-7 (*p* = 0.0638), it did not reach statistical significance. For all gene expression changes, *n* = 5-7 per group; significance indicated as *p < 0.05.

The involvement of immune cells in DSS-induced colitis between *Tspo* ^−/−^ and *Tspo* ^*fl/fl*^ mice was evaluated via a variety of markers. At baseline, expression of *Cd34*, a marker of mature mast cells, was significantly elevated in *Tspo* ^−/−^ mice compared to *Tspo* ^*fl/fl*^ mice, perhaps indicating a larger colonic mast cell population (Figure 4A). However, *Cd34* levels were not different between *Tspo* ^−/−^ and *Tspo* ^*fl/fl*^ colons during the course of DSS-induced UC. Mast cell tryptase (*Mcp6*) levels were elevated at both baseline and after DSS-induced colitis in *Tspo* ^−/−^ mice compared to *Tspo* ^*fl/fl*^ mice (Figure 4B), indicative of increased activation of mast cells in *Tspo* ^−/−^ colons. Expression of *Cd11c*, a marker of macrophages and dendritic cells, was increased at day-4 of UC colitis in *Tspo* ^−/−^ compared to *Tspo* ^*fl/fl*^ colons (Figure 4C). This was also indicative of differences in *Cd11c* expression across different UC timepoints within each genotype, in that *Tspo* ^−/−^ colons had an earlier peak in *Cd11c* expression, with significant up-regulation at day-4 compared to day-0 (*p* = 0.0132). In contrast, *Tspo* ^*fl/fl*^ colons showed no significant difference between baseline and day-4 levels of *Cd11c* (*p* = 0.0706). Expression of F4/80, the macrophage surface adhesion protein, as well as *Mcp1*, indicative of macrophage activation, and *Tnfα*, a pro-inflammatory cytokine, progressively increased throughout DSS-induced colitis, with no significant difference in levels between *Tspo* ^−/−^ and *Tspo* ^*fl/fl*^ colons (Figures 4D–F).

### 3.4 Baseline shifts in gene expression in *Tspo* ^−/−^ mice

**FIGURE 5:**
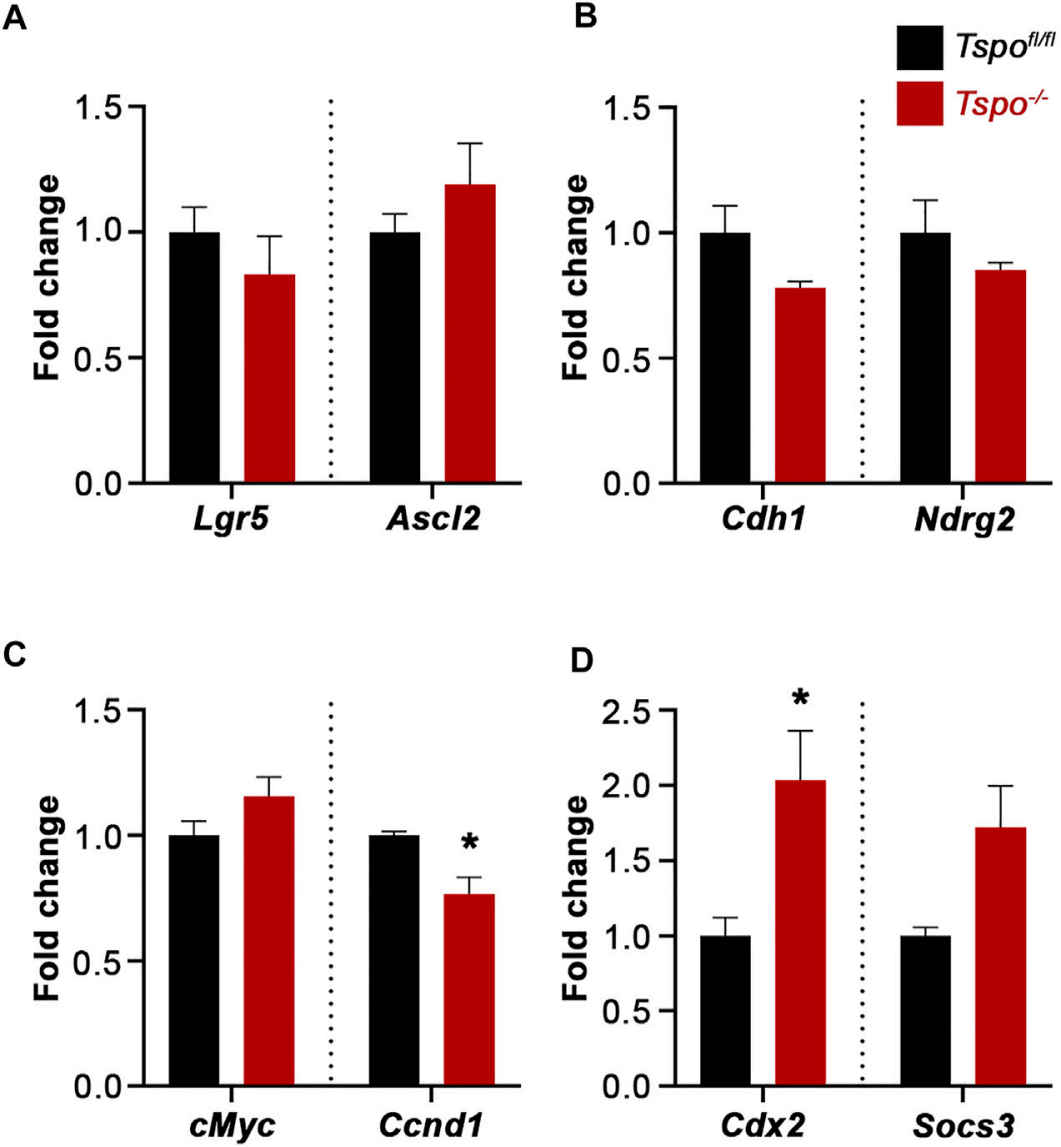
Baseline gene expression evaluating colonic epithelial functions in *Tspo* ^−/−^ mice. **(A)** Expression of *Lgr5* and *Ascl2*, indicative of intestinal stem cell identity, showed no difference between *Tspo* ^−/−^ and *Tspo* ^*fl/fl*^ colons. **(B)** Expression of *Cdh1* and *Ndrg2*, indicative of adhesion/epithelial integrity, showed no difference between *Tspo* ^−/−^ and *Tspo* ^*fl/fl*^ colons. **(C)** Expression of *cMyc* and *Ccnd1*, *Wnt* targets indicative of proliferation showed divergent effects; *cMyc* showed no difference between *Tspo* ^−/−^ and *Tspo* ^*fl/fl*^ colons, whereas *Ccnd1* was significantly downregulated in *Tspo* ^−/−^ colons (*p* = 0.0280). **(D)** Expression of *Cdx2*, transcription factor associated with intestinal epithelial development/identity, was significantly higher in *Tspo* ^−/−^ colons compared to *Tspo* ^*fl/fl*^ colons (*p* = 0.044). Expression of *Socs3*, a regulator of cytokine signaling in inflammation, was higher on an average in *Tspo* ^−/−^ colons compared to *Tspo* ^*fl/fl*^ colons, but did not reach statistical significance (*p* = 0.0625). Significance indicated as *p < 0.05.

Additional genes were examined to evaluate baseline differences in expression between *Tspo* ^−/−^ and *Tspo* ^*fl/fl*^ colons. There was no significant difference in expression of Leucine-rich repeat-containing G-protein coupled receptor 5 (*Lgr5*, a receptor seen in epithelial stem cells) and Achaete-scute-like 2 (*Ascl2,* a transcription factor that manages stem cell fate), suggesting that undifferentiated intestinal stem cell populations are similar between *Tspo* ^−/−^ and *Tspo* ^*fl/fl*^ colons (Figure 5A). Expression of Cadherin 1 (*Cdh1*, an epithelial tight junctional protein) and N-myc downstream regulated gene 2 (*Ndrg2*, a regulator of gut epithelial permeability) also remained unchanged between *Tspo* ^−/−^ and *Tspo* ^*fl/fl*^ colons, suggesting no obvious perturbations to the setup of epithelial integrity (Figure 5B). Of the proliferation markers, expression of cellular myelocytomatosis (*cMyc*, a cell proliferation indicator) was not different between *Tspo* ^−/−^ and *Tspo* ^*fl/fl*^ colons; however, expression of Cyclin D1 (*Ccnd1*, relevant to cell cycle progression) was significantly decreased in *Tspo* ^−/−^ colons. This suggested that the rate of intestinal epithelial turnover could be modestly decreased in *Tspo* ^−/−^ colons (Figure 5C). Expression of Caudal-type homeobox 2 (*Cdx2*, an epithelium-restricted transcription factor in the intestine), known to be responsible for intestinal identity and regulating immune cell infiltration, was significantly up-regulated in *Tspo* ^−/−^ compared to *Tspo* ^*fl/fl*^ colons (Figure 4D). Expression of Suppressor of cytokine signaling 3 (*Socs3*, a regulator of cytokine signaling and inflammation), albeit not different between *Tspo* ^−/−^ and *Tspo* ^*fl/fl*^ colons, showed a trend of increased baseline expression in *Tspo* ^−/−^ colons (Figure 5D).

**FIGURE 6:**
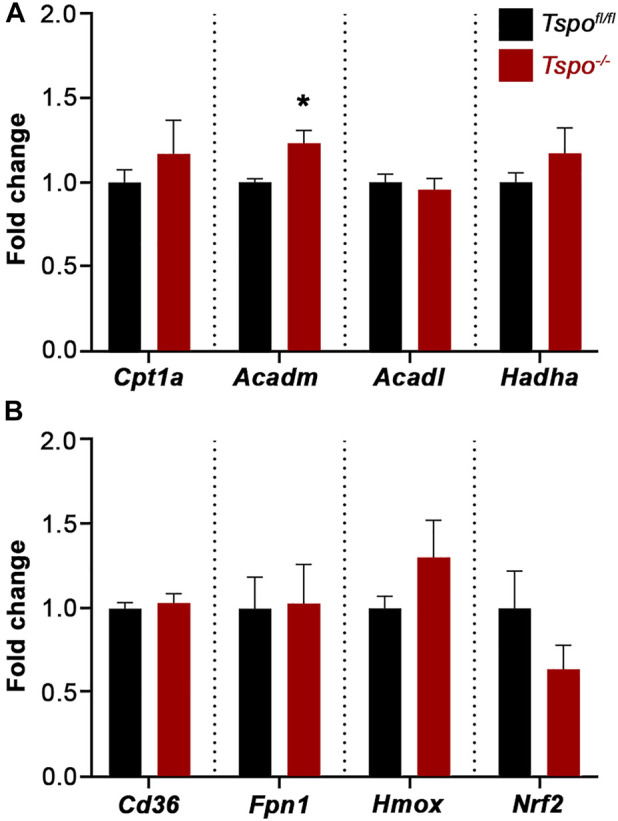
Baseline gene expression evaluating regulators of fatty acid oxidation and oxidative stress in *Tspo* ^−/−^ colons. **(A)** Expression of elements that indicate and/or regulate oxidative stress such as *Cd36*, *Fpn1*, *Hmox*, and *Nrf2*, showed no difference between *Tspo* ^−/−^ and *Tspo* ^*fl/fl*^ colons. **(B)** Expression of *Acadm*, a mitochondrial enzyme essential to metabolize medium chain fatty acids, was significantly higher in *Tspo* ^−/−^ colons compared to *Tspo* ^*fl/fl*^ colons (*p* = 0.0425). Expression of other elements associated with mitochondrial fatty acid β-oxidation such as *Cpt1a*, *Acadl*, and *Hadha*, showed no difference between *Tspo* ^−/−^ and *Tspo* ^*fl/fl*^ colons. Significance indicated as *p < 0.05.

In examining genes associated with an intestinal oxidative stress response: *Cd36* (involved in the uptake of oxidized lipids), Ferroportin 1 (*Fpn1,* regulates iron homeostasis and the redox state), Heme oxygenase 1 (*Hmox1,* catalyzes degradation of heme and a sensitive indicator of oxidative stress), and Nuclear factor erythroid 2-related factor 2 (*Nrf2*, a transcriptional regulator of antioxidant and cellular protective genes) were not significantly affected in baseline *Tspo* ^−/−^ compared to *Tspo* ^*fl/fl*^ colons (Figure 6A). Expression of genes associated with mitochondrial fatty acid oxidation indicated that Acyl-CoA dehydrogenase medium chain (*Acadm,* catalyzes the initial step in the mitochondrial β-oxidation of medium chain fatty acids), was significantly increased in *Tspo* ^−/−^ compared to *Tspo* ^*fl/fl*^ colons (Figure 6B). Other genes involved in this pathway such as Carnitine palmitoyl transferase (*Cpt1a*, essential for transport of long-chain fatty acids into the mitochondria for fatty acid oxidation), Acyl-CoA dehydrogenase long-chain (*Acadl,* catalyzes the initial step in the mitochondrial β-oxidation of long chain fatty acids) and Hydroxyacyl-CoA dehydrogenase subunit α (*Hadha,* catalyzes the last three steps in the mitochondrial β-oxidation of the long-chain fatty acids) were not significantly different between *Tspo* ^−/−^ and *Tspo* ^*fl/fl*^ colons (Figure 6B).

## 4 Discussion

In using *Tspo* ^−/−^ mice to investigate the mechanism and test the foundational value of TSPO-targeting pharmacological agents in UC, we uncover a functional link that corroborates TSPO involvement in the pathological basis of the disease. The DSS-induced colitis model has been shown to primarily induce epithelial barrier disruption, with inflammation only secondary to loss of epithelial integrity (Cooper et al., 1993; Laroui et al., 2012). This model yields colonic pathology that closely mimics that of human patients with UC (Wirtz et al., 2007). In this study, TSPO deficiency substantially exacerbated the inflammatory pathology associated with DSS-induced colitis. Although the impact of local versus global TSPO loss-of-function remains difficult to delineate, the pathologic findings provide conclusive evidence that effects specific to TSPO can influence inflammatory injury in colon. Physiologic TSPO function appears to offer a significant level of protection against colonic insults that might induce inflammation and injury. As TSPO expression is observed both in the intestinal epithelium and immune cells, we believe the exacerbated UC in *Tspo* ^−/−^ mice results from a combination of vulnerability in the epithelial barrier and dysregulation in specific immune responses.

The exacerbated UC phenotype in *Tspo* ^−/−^ mice became obvious as our pilot experiments to generate a colon cancer model using DSS and azoxymethane (Thaker et al., 2012) resulted in complete mortality in the *Tspo* ^−/−^ group during the second cycle of treatment (unpublished observations). During the 7-day DSS treatment in this study, we observed progressively aggravated clinical signs and body weight loss in *Tspo* ^−/−^ mice compared to *Tspo* ^*fl/fl*^ controls. The aggravated response in *Tspo* ^−/−^ mice was consistent with known pathological parameters of severity such as progressive, continuous ulceration of the colonic epithelium, edema, and shortened colon length (Okayasu et al., 1990). The severity of UC pathology *Tspo* ^−/−^ mice was also indicative of a degree of unrecoverable loss of intestinal structure.

As TSPO is expressed in the colonic epithelial cells, we first considered the possibility that specific loss of function in this cell type might be linked to a local predisposition to injury in *Tspo* ^−/−^ colons. Our finding that *Krt18*, the type I intermediate filament associated with the colonic epithelium, significantly decreases with UC in *Tspo* ^−/−^ mice was a clear indication of higher levels of colonic epithelial loss compared to *Tspo* ^*fl/fl*^ cohorts. Epithelial denudation has been correlated to the severity of DSS-induced UC in human patients (Corfe et al., 2015), and in our study, indicated the increased severity of UC pathology in *Tspo* ^−/−^ mice. In addition to serving as a marker, mutations in *Krt18* that disrupt the intermediate filament networks have also been shown to functionally increase epithelial permeability in human colonocytes (Zupancic et al., 2014). Keratin downregulation has also shown to dramatically increase susceptibility to cell death mediated by inflammatory cytokines such as TNF⍺ (Caulin et al., 2000). Supporting our gene expression results, substantial epithelial loss could be visualized in histopathology of *Tspo* ^−/−^ colons. Excessive inflammation seen with UC in *Tspo* ^−/−^ mice compared to *Tspo* ^*fl/fl*^ cohorts could be a consequence of amplified disruption of the epithelial barrier.

High-affinity benzodiazepine binding sites indicative of TSPO expression have also been identified in mast cells (Miller et al., 1988). Rats deficient in mast cells fail to develop clinical signs of DSS-induced colitis, indicating a crucial role for mast cells in UC pathogenesis (Araki et al., 2000). Moreover, it has been demonstrated that mast cell activation, identified by tryptase levels, has an essential proinflammatory role in triggering DSS-induced UC (Hamilton et al., 2011). Mast cell tryptase can also act as a trigger for other mediators of inflammation (Compton et al., 1998; Huang et al., 1998), potentially contributing to UC pathology. Functionally, different drugs that bind TSPO have been shown to have varying effects on mast cell degranulation (Miller et al., 1988; Suzuki-Nishimura et al., 1989; Sano et al., 1990; Yousefi et al., 2013). Investigating whether *Tspo* deletion affected the mast cell response, we identified significant upregulation of murine *Mcp6* (which is similar to the human mast cell hTryptase-β) in *Tspo* ^−/−^ colons. This can be considered an activation response, as *Cd34* expression indicative of mature mast cells (Drew et al., 2002), albeit increased in *Tspo* ^−/−^ colons, was not significantly different between subsequent (day-4 and day-7) timepoints between the two genotypes. It has been shown that *Mcp6* deficiency (Hamilton et al., 2011), or tryptase inhibition (Tremaine et al., 2002; Liu et al., 2021), could reduce UC pathology and intestinal fibrosis. In parallel, degranulation of mast cells has also been associated with the release of proinflammatory TNF⍺ (Gordon and Galli, 1991; Bischoff et al., 1999). We observed higher levels of *Tnfα* expression during UC in *Tspo* ^−/−^ colons, although this difference was not statistically significant. In forms of mast cell driven colitis, TNF⍺-driven effects have been demonstrated to be crucial for UC pathology (Rijnierse et al., 2006). It has also been shown that TNFα and other proinflammatory cytokines could persist in the colon at high levels for at least 14 days after DSS withdrawal (Yan et al., 2009). Therefore, protracted effects on inflammation could also be anticipated in *Tspo* ^−/−^ mice driven by both the inflammatory milieu and the degree of injury sustained.

We also observed that *Cd11c* levels peaked at day-4 of UC in *Tspo* ^−/−^ colons, which was significantly different and earlier than that measured for UC in *Tspo* ^*fl/fl*^ colons. *Cd11c* is expressed on the surface of monocytes, macrophages, and dendritic cells in the intestinal mucosa; these cells are important players in immune recognition and tolerance to the gut microbiota (Bain and Mowat, 2014). Increased infiltration of CD11c^+^ macrophages has been demonstrated to trigger mucosal injury in UC (Bernardo et al., 2018; Fuke et al., 2018), while reduction in CD11c^+^ macrophages ameliorated mucosal pathology (Arnold et al., 2015). Earlier up-regulation of *Cd11c* in *Tspo* ^−/−^ animals may reflect and/or influence the rapid onset and severity of colonic inflammation. In addition, CD11c^+^ phagocytes may also increase gut mucosal epithelial permeability (Ren et al., 2017), further contributing to colonic pathology through increased bacterial translocation across the gut barrier.

The upregulation of baseline colonic *Cdx2* expression in *Tspo* ^−/−^ mice indicates that TSPO deficiency contributed to a homeostatic shift, perhaps compensating for decreased epithelial barrier function. *Cdx2* gene-deleted heterozygous (*Cdx2* ^*+/−*^) mice showed hypersensitivity to DSS-induced acute UC and increased permeability of the colonic epithelium (Calon et al., 2007). *Cdx2* is known to control cell-cell adhesion and extracellular matrix composition/interactions (Lorentz et al., 1997); baseline *Cdx2* upregulation may serve as a compensatory mechanism to offset increased vulnerability to epithelial permeability in *Tspo* ^−/−^ mice. High expression of TNFα, through NF-κB and p38 MAPK pathways, could downregulate *Cdx2* (Coskun et al., 2014), suggesting that there might be significant cross-talk between epithelium-specific and different immune pathways.

There also exists cross-talk between *Cdx2* and *Wnt* signaling, regulating the proliferative compartment of normal intestinal crypts (Guo et al., 2010). Our finding that the *Wnt* target *Ccnd1* was decreased in *Tspo* ^−/−^ colons might indicate a lower capacity for epithelial proliferation and turnover, potentially affecting regeneration after ulcerative injury. However, based on similar *Lgr5* and *Ascl2* levels between *Tspo* ^*fl/fl*^ and *Tspo* ^−/−^ colons, stem cell populations and adhesion molecule expression were not altered in *Tspo* ^−/−^ colons. Although *Cd34* expression also believed to be associated with these adult stem cells (Stzepourginski et al., 2017), are higher at baseline in *Tspo* ^−/−^ colons, the differences are in all probability linked to mature mast cell numbers (consistent with higher baseline *Mcp6* expression levels). Although stem cell populations are similar, given the progressive severity of UC pathology *Tspo* ^−/−^ mice, it is plausible that TSPO deficiency might also negatively impact epithelial recovery and functional restoration in the colon. This speculation is supported by pharmacology that TSPO binding drugs, PK11195 and Ro5-4864 could accelerate recovery after DSS-induced UC (Ostuni et al., 2010). Although the extent of TSPO-mediated action remains unclear, positive and negative outcomes in DSS-induced UC have been reported for relatively low-affinity TSPO binding molecules such as curcumin (Li et al., 2013; Liu et al., 2013; Guo et al., 2022) and retinoic acid (Oehlers et al., 2012; Li et al., 2013; Rampal et al., 2021).

As recent developments on TSPO function have indicated possible roles in regulation of mitochondrial fatty acid oxidation (Tu et al., 2016) and oxidative stress responses (Gatliff et al., 2014), we examined genes associated with these functions in *Tspo* ^−/−^ colons. The finding that *Acadm* is up-regulated in *Tspo* ^−/−^ colons is consistent with evidence that there exist subtle metabolic shifts with TSPO loss-of-function. In tissues active in lipid metabolism that show very high expression of TSPO, we have previously reported a functional up-regulation of mitochondrial fatty acid oxidation, and associated increase in gene expression of *Cpt1a, Acadm, Acadl*, and *Hadha* (Tu et al., 2016). Increases in *Cpt1a, Acadl* and *Hadha* were not observed in *Tspo* ^−/−^ colons; therefore, it is conceivable that the present study’s use of entire colons for gene expression assays, as opposed to just TSPO-expressing epithelial layers or mast cells, could have diluted any cell-type specific phenotypes. With regard to oxidative stress, direct evidence previously demonstrated that TSPO could stabilize mitochondrial architecture during inflammatory stress in colonic cells (Issop et al., 2016). We have previously observed that TSPO involvement in oxidative stress is cell-type dependent (Tu et al., 2016; Zhao et al., 2016). Although we did not observe any baseline perturbations in gene expression associated with oxidative stress responses in *Tspo* ^−/−^ colons, baseline expression may not reflect active inflammatory cell states associated with UC.

In summary, despite lack of a clear structure-function relationship for the different TSPO-mediated effects that are described in the literature (Selvaraj et al., 2015; Selvaraj and Stocco, 2015), and mechanisms that delineate TSPO pharmacology (Gavish et al., 1999; Gavish and Veenman, 2018), our findings in this *Tspo* ^−/−^ model demonstrates a protective effect for TSPO on the colonic epithelium and confirms that there might be potential for therapeutic interventions with ligands that enhance TSPO function. For example, the TSPO-binding benzodiazepine derivatives Ro5-4864 (4'-chlorodiazepam) and flunitrazepam, which were both shown to quickly suppress mast cell activation *in vivo* and *in vitro* (Yousefi et al., 2013), may be considered to be TSPO agonists. Understandably, similar actions albeit with differing efficacies, occur for these same drugs in the *in vivo* DSS-induced colitis model (Ostuni et al., 2010). The hypothesis that TSPO likely provides a dual therapeutic effect, involving both epithelial barrier-specific functions and immune-cell mediated pathways, is an attractive mechanism that merits more detailed investigation. Fundamentally, we uncover that TSPO deletion/loss-of-function, albeit inconsequential at a resting/healthy state, makes mice more vulnerable to insults. Future studies that dissect the precise molecular function of TSPO might help describe its physiological and pharmacological significance in UC and other inflammatory disease models.

## Data Availability

The original contributions presented in the study are included in the article/Supplementary Material; further inquiries can be directed to the corresponding author.
