# Supplementary material for: Mitochondrial translocator protein deficiency exacerbates pathology in acute experimental ulcerative colitis
Source: Front Physiol. 2022 Aug 19;13:896951. doi: 10.3389/fphys.2022.896951 (PMC9437295; doi:10.3389/fphys.2022.896951)
Supplement: Supplementary file 1 [file DataSheet1.PDF]

SUPPLEMENTARY TABLES

Table S1. Primers used for quantitative PCR assays

Target transcript	Primer
Tnfα	TaqMan Assay ID: Mm00443260_g1
F4/80	TaqMan Assay ID: Mm00802529_m1
Cd34	TaqMan Assay ID: Mm00519283_m1
Gapdh	TaqMan Assay ID: Mm99999915_g1
Cd11c	5’- CTGGATAGCCTTTCTTCTGCTG – 3’<br>5’ – GCACACTGTGTCCGAACTCA – 3’
Krt18	5’ – CAAGTCTGCCGAAATCAGGGAC – 3’<br>5’ – TCCAAGTTGATGTTCTGGTTT – 3’
Mcp1	5’ – TTAAAAACCTGGATCGGAACCAA – 3’<br>5’ – GCATTAGCTTCAGATTTACGGGT – 3’
Mcp6	5’ – CATTTCTGCGGAGGTTCTCTC – 3’<br>5’ – CACCACGATCCTGTTCAAAGA – 3’
Cpt1a	5′- CTCAAACCTATTCGTCTTCTG-3′<br>5’- TTGGATGGTGTCTGTCTC-3’
Acadm	5’- AACACAACACTGGAAAGCGG-3’<br>5’- TTCTGCTGTTCCGTCAACTCA-3’
Acadl	5’- TTTCCTCGGAGCATGACATTT-3’<br>5’- GCCAGCTTTTTCCCAGACCT-3’
Hadha	5’- TGCATTTGCCGCAGCTTTAC-3’<br>5’- GTTGGCCCAGATTTCGTTCA-3’
Cd36	5’- GATGACGTGGCAAAGAACAG-3’<br>5’- CAGTGAAGGCTCAAAGATGG-3’
Fpnl	5’- ACCAAGGCAAGAGATCAAACC-3’<br>5’- AGACACTGCAAAGTGCCACAT-3’
Hmox	5’- AAGCCGAGAATGCTGAGTTCA-3’<br>5’- GCCGTGTAGATATGGTACAAGGA-3’
Nrf2	5’- CCTCCATTCAGTAACAACTGGAC-3’<br>5’- GCCAAACTTGCTCCATGTCC-3’
Cdh1	5’- CTCCAGTCATAGGGAGCTGTC-3’<br>5’- TCTTCTGAGACCTGGGTACAC-3’
Ndrg2	5’- TGTTGCCAGGACAAACACCC-3’<br>5’- AAAAGTGACCGAGCCATAAGG-3’
Lgr5	5’- CAGCCTCAAAGTGCTTATGCT-3’<br>5’- GTGGCACGTAACTGATGTGG-3’
Ascl2	5’- CCGTGAAGGTGCAAACGTC-3’<br>5’- CCCTGCTACGAGTTCTGGTG-3’

cMyc	5’- ATGCCCCTCAACGTGAACTTC-3’<br>5’- GTCGCAGATGAAATAGGGCTG-3’
Ccnd1	5’- ACCTCCCGCAGTGTTCCTATT-3’<br>5’- CACAGACCTCTAGCATCCAGG-3’
Cdx2	5’- CAAGGACGTGAGCATGTATCC-3’<br>5’- GTAACCACCGTAGTCCGGGTA-3’
Soc3	5’- CCCTTGCAGTTCTAAGTTCAACA-3’<br>5’- ACCTTTGACAAGCGGACTCTC-3’
Tbp	5’- CCTTGTACCCTTCACCAATGAC-3’<br>5’- ACAGCCAAGATTCACGGTAGA-3’
